# Association of 25-Hydroxyvitamin D status and genetic variation in the vitamin D metabolic pathway with FEV_1_ in the Framingham Heart Study

**DOI:** 10.1186/s12931-015-0238-y

**Published:** 2015-07-01

**Authors:** JG Hansen, W Gao, J Dupuis, GT O’Connor, W Tang, M Kowgier, A Sood, SA Gharib, LJ Palmer, M Fornage, SR Heckbert, BM Psaty, SL Booth, SUNLIGHT Consortium, Patricia A Cassano

**Affiliations:** Division of Nutritional Sciences, Cornell University, 209 Savage Hall, Ithaca, NY 14853 USA; Department of Biostatistics, Boston University School of Public Health, Boston, MA USA; The National Heart, Lung, and Blood Institute’s Framingham Heart Study, Framingham, MA USA; Pulmonary Center, Department of Medicine, Boston University School of Medicine, Boston, MA USA; Ontario Institute for Cancer Research, Toronto, ON Canada; Dalla Lana School of Public Health, University of Toronto, Toronto, ON Canada; University of New Mexico, Albuquerque, NM USA; Computational Medicine Core, Center for Lung Biology, Division of Pulmonary & Critical Care Medicine, Department of Medicine, University of Washington, Seattle, WA USA; School of Public Health, University of Adelaide, Adelaide, Australia; Institute of Molecular Medicine, University of Texas Health Science Center at Houston, Houston, TX USA; Human Genetics Center, School of Public Health, University of Texas Health Science Center at Houston, Houston, TX USA; Cardiovascular Health Research Unit, University of Washington, Seattle, WA USA; Department of Epidemiology, University of Washington, Seattle, WA USA; Group Health Research Institute, Group Health Cooperative, Seattle, WA USA; Department of Medicine, University of Washington, Seattle, WA USA; Jean Mayer USDA Human Nutrition Research Center on Aging, Tufts University, Boston, MA USA; The SUNLIGHT Consortium (Study of Underlying Genetic Determinants of Vitamin D and Highly Related Traits), ᅟ, ᅟ; Division of Biostatistics and Epidemiology, Department of Healthcare Policy and Research, Weill Cornell Medical College, New York, NY USA

**Keywords:** Vitamin D, 25-hydroxyvitamin D, FEV_1_, *CYP2R1*, Lung function, Framingham Heart Study

## Abstract

**Background:**

Vitamin D is associated with lung function in cross-sectional studies, and vitamin D inadequacy is hypothesized to play a role in the pathogenesis of chronic obstructive pulmonary disease. Further data are needed to clarify the relation between vitamin D status, genetic variation in vitamin D metabolic genes, and cross-sectional and longitudinal changes in lung function in healthy adults.

**Methods:**

We estimated the association between serum 25-hydroxyvitamin D [25(OH)D] and cross-sectional forced expiratory volume in the first second (FEV_1_) in Framingham Heart Study (FHS) Offspring and Third Generation participants and the association between serum 25(OH)D and longitudinal change in FEV_1_ in Third Generation participants using linear mixed-effects models. Using a gene-based approach, we investigated the association between 241 SNPs in 6 select vitamin D metabolic genes in relation to longitudinal change in FEV_1_ in Offspring participants and pursued replication of these findings in a meta-analyzed set of 4 independent cohorts.

**Results:**

We found a positive cross-sectional association between 25(OH)D and FEV_1_ in FHS Offspring and Third Generation participants (*P* = 0.004). There was little or no association between 25(OH)D and longitudinal change in FEV_1_ in Third Generation participants (*P* = 0.97). In Offspring participants, the *CYP2R1* gene, hypothesized to influence usual serum 25(OH)D status, was associated with longitudinal change in FEV_1_ (gene-based *P* < 0.05). The most significantly associated SNP from *CYP2R1* had a consistent direction of association with FEV_1_ in the meta-analyzed set of replication cohorts, but the association did not reach statistical significance thresholds (*P* = 0.09).

**Conclusions:**

Serum 25(OH)D status was associated with cross-sectional FEV_1_, but not longitudinal change in FEV_1_. The inconsistent associations may be driven by differences in the groups studied. *CYP2R1* demonstrated a gene-based association with longitudinal change in FEV_1_ and is a promising candidate gene for further studies.

**Electronic supplementary material:**

The online version of this article (doi:10.1186/s12931-015-0238-y) contains supplementary material, which is available to authorized users.

## Introduction

Decreased lung function due to airflow obstruction is the primary characteristic of chronic obstructive pulmonary disease (COPD), the 3rd leading cause of mortality in the United States [1]. Vitamin D status, assessed by the circulating serum biomarker 25-hydroxyvitamin D [25(OH)D], plays a well-known role in bone health, and is also associated with non-skeletal outcomes including lung function [2, 3]. National surveys estimate that over 30 % of Americans are at risk for vitamin D insufficiency (defined as serum 25(OH)D <20 ng/mL) [3, 4]. Vitamin D is obtained through sun exposure and diet [2], and genome-wide association studies (GWAS) have identified single nucleotide polymorphisms (SNPs) in vitamin D metabolic genes that are significantly associated with serum 25(OH)D concentrations [5, 6].

The active vitamin D metabolite, 1,25(OH)_2_D, is constitutively synthesized from 25(OH)D *in vitro* in renal and extra-renal tissues including lung tissue [7] and is involved in biological processes critical to lung function including inflammation and airway remodeling [8–10]. Several cross-sectional, population-based observational studies have demonstrated strong, positive associations between vitamin D levels and lung function [11–13], although one study in the Hertfordshire cohort did not replicate cross-sectional associations [14]. Additionally, vitamin D deficiency is common in COPD patients [15], and high-dose vitamin D supplementation reduced COPD exacerbations in patients with severe vitamin D deficiency [16]. High vitamin D levels have also been associated with reduced risk of respiratory infections [13, 17], although randomized controlled trials of vitamin D supplementation and respiratory infections have been inconclusive [18, 19]. A recent cohort study demonstrated that low serum vitamin D was associated both with steeper lung function decline and a higher risk of developing COPD [20]. Additionally, an observational study in COPD patients reported no association between serum 25(OH)D and longitudinal lung outcomes [21], but a recent population-based study in an elderly male cohort reported steeper lung function decline in current smokers with serum 25(OH)D ≤20 ng/mL versus smokers with higher 25(OH)D [22]. Genetic variants in the vitamin D binding protein, encoded by the *GC* gene, are associated with COPD risk [15, 23–26], and *GC* may be an important mediator of hypothesized vitamin D effects on lung function [27].

Our study investigated the association of serum 25(OH)D with lung function in two generational cohorts of the Framingham Heart Study (FHS). Both serum measurements of vitamin D and genetic variants associated with serum vitamin D levels were investigated in association with cross-sectional FEV_1_ and rate of change in FEV_1_. Overall, this study provides a comprehensive exploration of serum 25(OH)D associations with FEV_1_ in a healthy, adult population-based sample including both men and women.

## Methods

### Study design

We investigated the association of serum 25(OH)D with lung function in two Framingham Heart Study (FHS) generational cohorts, the Offspring cohort and the Third Generation cohort. The availability of the specific data needed for the stages of the research was the main driver of which cohort(s) contributed to each analysis. We pursued replication of the SNP findings in four independent epidemiologic cohort studies, namely the Health, Aging and Body Composition Study (Health ABC), the Coronary Artery Risk Development in Young Adults Study (CARDIA), the Busselton Health Study (BHS), and the Cardiovascular Health Study (CHS), and we investigated the SNPs in relation to serum 25(OH)D status in the SUNLIGHT consortium [6].

### Study population and ethics

Study participants were from the Offspring and Third Generation cohorts of the FHS, a longitudinal family-based study established in 1948 in Framingham, MA. The Offspring cohort, consisting of original cohort offspring and their spouses, began in 1971 [28]. The Third Generation cohort was initiated in 2002, enrolling children of the Offspring cohort [29]. Self-reported ethnicity across all FHS cohorts was >99 % Caucasian [29].

Third Generation participants with serum 25(OH)D measurements from Exam 1 (2002–2005) and spirometry measurements from Exams 1 and 2 (2008–2010) were included in all serum 25(OH)D—FEV_1_ analyses (*N* = 3,599; 88 % of full cohort). 1,435 Offspring participants (28 % of full cohort) had serum 25(OH)D and spirometry measurements at either Exam 6 (1995–1998) or 7 (1998–2001), and were included in the cross-sectional serum 25(OH)D—FEV_1_ analyses. The Offspring participants were not included in the serum 25(OH)D—rate of change in FEV_1_ analysis because only 24 % of the full Offspring cohort had repeated lung function measured after serum vitamin D, and we expected survivor bias to affect the longitudinal associations (see Results section). Serum vitamin D and pulmonary function were measured concurrently in Offspring participants, and thus the Offspring cohort data contributed to estimation of the cross-sectional association. If any bias exists, it is expected to be conservative (estimate closer to the null value) because the range in vitamin D may be restricted in the subsample with data. The SNP—rate of change in FEV_1_ associations were studied in 3,230 Offspring participants (63 % of full cohort) with GWAS genotype data and spirometry measurements spanning over Exams 5–8, which provided optimal information on longitudinal change in lung function given the number of repeated measurements and the length of time between measurements.

All study participants provided written informed consent for this study, and local institutional review boards approved the study protocols.

### Measures

Genotyping was performed using the Affymetrix 500 K SNP array with a supplemental Affymetrix 50 K gene-focused array. Genotyping and imputation methods are described in detail elsewhere [6, 30]. 241 imputed SNPs in six candidate genes with well-established roles in vitamin D metabolism and transport [*CYP24A1, CYP27A1, CYP27B1, CYP2R1, DHCR7/NADSYN1* (these two genes considered jointly as a candidate genomic locus due to prior GWAS associations [5, 6])*,* and *GC*] were analyzed (+/− 5 KB region on either end of genes included; Additional file 1: Table S1 for details).

Pulmonary function test measurements meeting American Thoracic Society/European Respiratory Society criteria for acceptability and reproducibility were used [31] (see Additional file 1 for further details).

Serum 25(OH)D was measured using radioimmunoassay (DiaSorin Inc, Stillwater, MN, USA) [6, 32, 33], and log-transformed values were used in all analyses (see Additional file 1 for further details). Offspring serum samples for 25(OH)D were collected between 1997–2001 [32] and Third Generation serum samples between 2001–2005 [6, 33]; assays were completed separately in different laboratories. However, all FHS samples were analyzed after 1998, so assay drifts due to the reformulated RIA assay, described for the NHANES data [34], are not expected to affect 25(OH)D measurements in Framingham (Additional file 1 for further quality control information on assays).

Smoking pattern was defined as: persistent smoker (current smoker, all time points during follow-up), intermittent smoker (current smoker at ≥ 1 time point), former smoker (former smoker, all time points), and never smoker (never smoker, all time points).

### Statistical analysis

All analyses used linear mixed effect models with a random effect to account for familial correlation. The independent variable was FEV_1_; in serum 25(OH)D—rate of change in FEV_1_ analyses, the coefficient of interest was the interaction of 25(OH)D x time (time = time elapsed between the initial FEV_1_ measurement and each subsequent measurement), which estimated the effect of serum 25(OH)D on rate of change in FEV_1_. The cross-sectional association of serum 25(OH)D with FEV_1_ was estimated by the coefficient for serum 25(OH)D from the mixed effect models; all participants with serum 25(OH)D had a concurrent measurement of pulmonary function. In SNP—rate of change in FEV_1_ analyses, the coefficient for the interaction of SNP x time estimated the additive effect of the coded allele on rate of change in FEV_1_. All models were adjusted for baseline age, sex, height, current smoking status, smoking pattern during follow-up (defined in previous paragraph), the interaction of smoking pattern x time, and baseline pack-years. Serum 25(OH)D—FEV_1_ models were further adjusted for month of 25(OH)D measurement, body mass index (BMI), and FHS cohort (for models including both Offspring and Third Generation participants). Genetic models were further adjusted for the first two ancestry principal components to account for population substructure [35]. Analyses were completed using R (version 2.15.3).

A staged approach was used for SNP—FEV_1_ analyses. First, a gene-based *P-*value was calculated for the association of each candidate gene with rate of change in FEV_1_ using the VEGAS program [36, 37]. VEGAS calculates a single, gene-based test statistic based on *P-*values from the SNP—rate of change in FEV_1_ regression analysis across a given gene region, taking the linkage disequilibrium (LD) structure of the gene into account [36]. A gene-based *P-*value < 0.05 was chosen to select genes to follow-up in replication. Second, the most significant SNP from candidate genes with a gene-based *P* < 0.05 was assessed for replication in a meta-analysis of the four replication cohorts. The most significant SNP from each gene with a gene-based *P* < 0.05 was further evaluated for association with serum 25(OH)D using data from the SUNLIGHT consortium, which comprised 33,996 individuals of European ancestry, including FHS participants, with GWAS data and the serum 25(OH)D phenotype [6].

## Results

Average serum 25(OH)D in the 28 % of the Offspring participants with data was 18.0 ng/mL, compared with 34.5 ng/mL in Third Generation participants; similarly, the proportion of participants at risk of vitamin D deficiency (defined as serum 25(OH)D <12 ng/mL) was 14.4 % and 1.2 % in the Offspring and Third Generation cohorts, respectively (Table 1). As expected, Offspring participants were older, had lower baseline FEV_1_, and were more likely to be former smokers than the Third Generation participants (Tables 1 and 2).

**Table 1:** Baseline^1^ population characteristics of Framingham Heart Study participants

25(OH)D—FEV_1_ Analyses	Offspring Cohort	Third Generation Cohort	*P*-value^2^
N in analysis	*N* = 1,435	*N* = 3,599	
FEV_1_, L	2.7 (0.8)	3.6 (0.8)	<0.0001
Follow-up duration, yr^3^	7.2 (1.9)	6.1 (0.6)	<0.0001
Baseline age, yr	59.9 (9.2)	40.2 (8.7)	<0.0001
Male, %	48	47	0.84
Height, cm	168.0 (9.1)	170.6 (9.3)	<0.0001
Baseline pack-years	26.0 (22.7)	13.7 (14.2)	<0.0001
Current smokers^4^, %	12.8	15.2	0.02
Former smokers^4^, %	50.8	27.0	<0.0001
BMI	28.0 (5.1)	26.9 (5.4)	<0.0001
25(OH)D, ng/ml^5^	18.0 (1.5)	34.5 (1.5)	<0.0001
N at risk of 25(OH)D deficiency (<12 ng/mL), %	207 (14.4 %)	44 (1.2 %)	<0.0001
N at risk of 25(OH)D inadequacy (<20 ng/mL), %	801 (55.8 %)	311 (8.6 %)	<0.0001

^1^Baseline measurements are from the exam at time of vitamin D measurement (either Exam 6 or 7 for Offspring, and Exam 1 for Third Generation); Participant characteristics listed as mean (SD) for continuous variables and number (%) for categorical variables

^2^ *P*-values for continuous variables calculated from unpaired *t*-test; *P*-values for categorical variables calculated from chi-square test

^3^Follow-up duration of spirometry measurements *subsequent* to serum 25(OH)D measurement; average duration calculated for *N* = 1,223 Offspring cohort participants with longitudinal spirometry and *N* = 2,894 Third Generation cohort participants

^4^Current smokers at baseline; former smokers at all time points

^5^Geometric mean of 25(OH)D

**Table 2:** Baseline^1^ population characteristics of Framingham Heart Study participants contributing to SNP analysis

SNP—FEV_1_ Analysis	Offspring Cohort
N in analysis	*N* = 3,230
FEV_1_, L	3.0 (0.8)
Follow-up duration, yr	14.7
Baseline age, yr	50.9 (10.3)
Male, %	47
Height, cm	165.5 (9.5)
Baseline pack-years	25.4 (21.3)
Current smokers^2^, %	24.6
Former smokers^2^, %	39.8

^1^ Baseline measurements refer to Exam 5; Participant characteristics listed as mean (SD) for continuous variables and number (%) for categorical variables

^2^ Current smokers at baseline; former smokers at all time points

### 25(OH)D associations with cross-sectional FEV_1_ and rate of change in FEV_1_

**Fig. 1:**
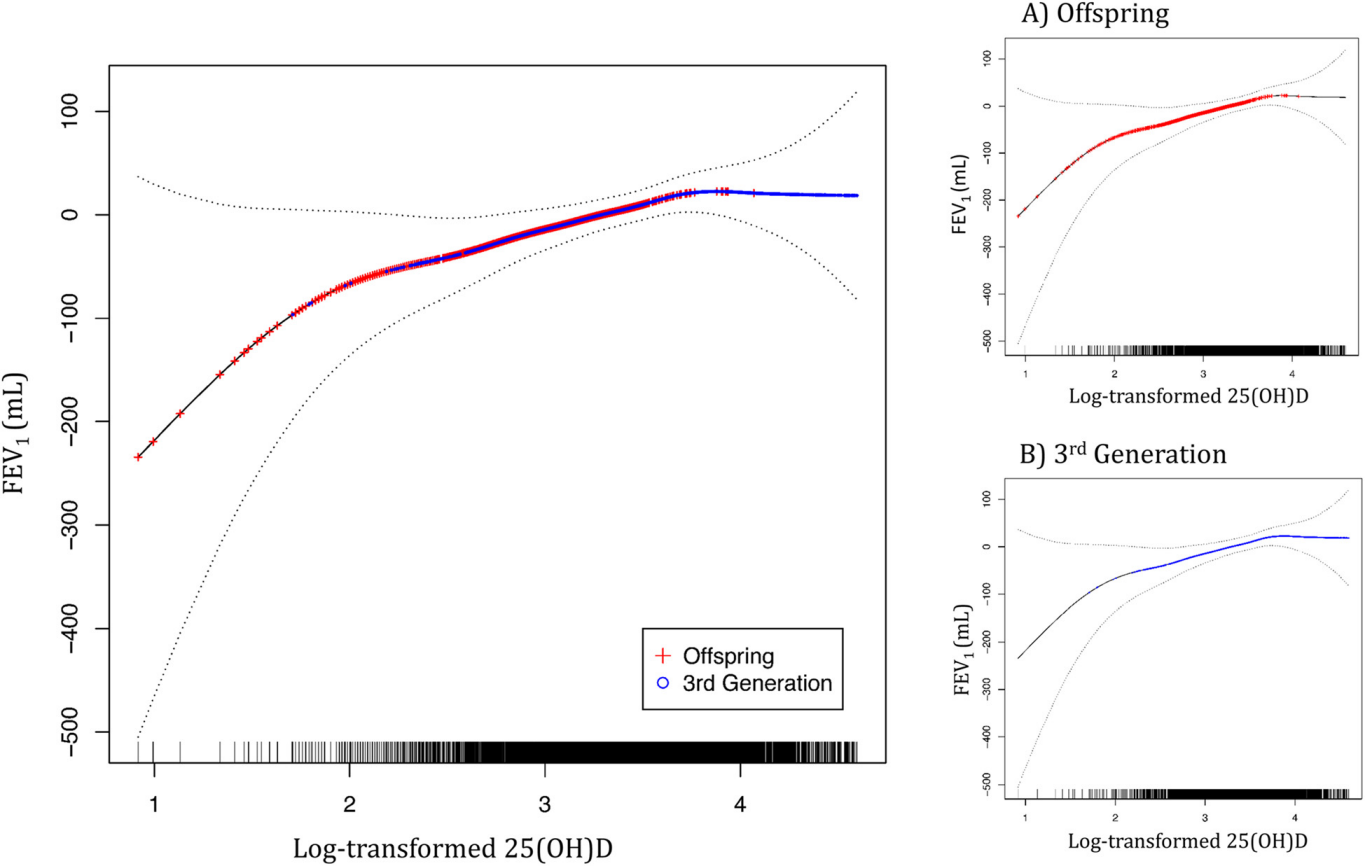
Spline analysis of log-transformed 25(OH) D by residual FEV_1_

25(OH)D was positively associated with FEV_1_ in the cross-sectional analysis of the combined sample of Offspring and Third Generation participants, such that a 1-unit increase in log-transformed 25(OH)D was associated with a 45 mL increase in FEV_1_ (*P* = 0.004) (Table 3). A consistent direction of association was observed for the dichotomous vitamin D variable at thresholds of <12 ng/mL or <20 ng/mL, using the Institute of Medicine thresholds for risk of vitamin D deficiency and inadequacy, respectively [3], but coefficients for the dichotomous serum vitamin D variables did not reach the significance threshold of *P* < 0.05. The spline analysis estimating the cross-sectional serum 25(OH)D—FEV_1_ association showed an approximately linear, positive association for serum 25(OH)D < 12 ng/mL. In the 12 to 40 ng/mL range, the serum 25(OH)D—FEV_1_ association attenuated, and a plateau was reached above a threshold of about 40 ng/mL (Fig. 1). The pattern of association did not differ between Offspring and Third Generation participants (sub panels of Fig. 1), and the estimates of the linear serum 25(OH)D—FEV_1_ association were about the same in both cohorts (beta coefficient for serum 25(OH)D—FEV_1_ association was 37 (SE: 29, *P* = 0.20) and 34 (SE: 19, *P* = 0.08) mL in Offspring and Third Generation, respectively).

**Table 3:** Cross-sectional multivariable association of 25(OH)D with baseline FEV_1_ (mL) in the Offspring and Third Generation cohorts, combined (*N* = 5,034)

Model categorization of vitamin D:	β	SE	*P*
Continuous : Log-transformed 25(OH)D	45.2	15.5	0.004
Dichotomy 1: At risk of vitamin D *deficiency* (<12 ng/mL) vs. not	−46.7	26.7	0.08
Dichotomy 2: At risk of vitamin D *inadequacy* (<20 ng/mL) vs. not	−30.6	16.6	0.07

Adjusted for: baseline age, sex, height, smoking pattern, current smoking status, baseline pack-years, FHS cohort, baseline BMI, and month of blood draw; all coefficients show expected direction of effect

*β* beta coefficient, *SE* standard error, *P* P-value

We found definitive evidence of survivor bias for the longitudinal analysis: the 24 % of Offspring cohort members with both serum 25(OH)D measurements and subsequent repeated FEV_1_ measurements had a much *lower* rate of change in FEV_1_ compared to the 63 % of Offspring cohort members with only longitudinal lung function data (see Additional file 1 for details). Thus, we estimated the serum 25(OH)D—rate of change in FEV_1_ association in Third Generation cohort participants only. There was little or no association between serum 25(OH)D and rate of change in FEV_1_ (*P* = 0.97; Table 4). Neither was there any statistical evidence for a differential effect of serum 25(OH)D on rate of change in FEV_1_ by smoking status.

**Table 4:** Association of 25(OH)D with rate of change in FEV_1_ (mL/yr) in the Third Generation cohort

	Third Generation Cohort		
	(*N* = 3,599)		
Model parameterization of vitamin D:	β	SE	*P*
Continuous : Log-transformed 25(OH)D	−0.06	1.7	0.97
Dichotomy 1: At risk of vitamin D *deficiency* (<12 ng/mL) vs. not^a^	−0.02	6.9	0.997
Dichotomy 2: At risk of vitamin D *inadequacy* (<20 ng/mL) vs. not	2.0	2.5	0.41

Adjusted for: baseline age, sex, height, smoking pattern over follow-up and its interaction with time, baseline pack-years, current smoking status at each time point, BMI, and month of blood draw

*β* beta coefficient for 25(OH)D x time effect, *SE* standard error, *P* P-value

^a^Interpretation: Third Generation participants at risk of vitamin D deficiency have a 0.02 mL/yr steeper rate of decline compared to Third Generation participants not at risk of deficiency

### Vitamin D metabolic gene SNPs and rate of change in FEV_1_

We explored the association of SNPs in select vitamin D metabolic genes with rate of change in FEV_1_ in Offspring participants, and two genes, namely *CYP27B1* and *CYP2R1,* were associated with rate of change in FEV_1_ at a nominal gene-based *P* < 0.05. The most significant SNP associations in *CYP27B1* and *CYP2R1* were for rs10877013 (*P* = 0.02) and rs11819875 (*P* = 0.004), respectively, and the minor alleles of these SNPs were associated with steeper FEV_1_ decline (Table 5). No other candidate genes met the threshold for gene-based statistical significance (*P* < 0.05), and thus none were considered further.

**Table 5:** Association of the most statistically significant SNP per gene with the rate of change in FEV_1_ in FHS and in the meta-analyzed replication cohorts

Gene	Gene-based *P* Value	Chr	Total SNPs in gene	Best SNP	Position	Coded Allele^a^	Freq	FHS (*N* = 3,230)			Meta-analyzed replication cohorts (*N* = 7,246)		
								β	SE	*P*	β	SE	*P*
*CYP27B1*	0.02248	12	5	rs10877013	56451352	T	0.30	−1.3	0.5	0.02	−0.4	0.5	0.40
*CYP2R1*	0.04417	11	15	rs11819875	14873873	G	0.18	−1.9	0.7	0.004	−1.0	0.6	0.09

Adjusted for: baseline age, sex, height, smoking pattern over follow-up and its interaction with time, baseline smoking pack-years, and the first two principal components for genetic ancestry

*Chr* chromosome, *SNP* single nucleotide polymorphism, *β* beta coefficient for SNP x time effect, *SE* standard error, *P* P-value

^a^Coded allele and frequency for the Framingham Heart Study (FHS). All effect estimates presented in terms of FHS coded allele. Coded allele frequencies between FHS and replication cohorts were nearly identical

We explored replication of the most significant SNP in *CYP27B1* and *CYP2R1* with change in FEV_1_ in a meta-analyzed set of four independent cohorts. Rs11819875 (*CYP2R1*) showed a consistent direction of association with FEV_1_ in FHS and the meta-analyzed set of replication cohorts, but this association was not replicated by statistical significance thresholds (β = −1.0, 95 % CI: −2.2, 0.2, *P* = 0.09; Table 5 and Additional file 1: Table S2). Rs11819875 was associated with a lower concentration of serum 25(OH)D in data from the SUNLIGHT consortium (*P* = 0.15 from combined SUNLIGHT discovery and replication cohorts). Rs10877013 in *CYP27B1* showed little to no association with rate of change in FEV_1_ in the meta-analyzed replication cohort at (β = −0.4, 95 % CI: −1.4, 0.6, *P* = 0.40; Table 4), and rs10877013 was associated with lower 25(OH)D in SUNLIGHT (*P* = 0.11 from combined SUNLIGHT discovery and replication cohorts).

## Discussion

In this population-based cohort study of adults, we investigated the association of vitamin D with cross-sectional FEV_1_ and longitudinal FEV_1_, considering both serum 25(OH)D status and genetic variants in selected vitamin D metabolic genes. We demonstrated a strong, positive cross-sectional association of serum 25(OH)D with FEV_1_, consistent with the findings of most previously published studies [11–13, 20]. However, serum 25(OH)D was not associated with longitudinal change in FEV_1_ in Third Generation participants. *CYP2R1*, a vitamin D metabolism gene hypothesized to influence usual 25(OH)D status, was associated with longitudinal change in FEV_1_ in a gene-based analysis, but the most significant SNP in this gene was not replicated in a meta-analyzed set of replication cohorts.

Our cross-sectional findings provide evidence that associations between 25(OH)D and lung function may be non-linear and limited to individuals with inadequate vitamin D status. The lack of association of serum 25(OH)D with longitudinal change in FEV_1_ may be explained by characteristics of the study population; the Third Generation cohort was comprised of middle-aged participants with excellent serum 25(OH)D status. More than 90 % of the Third Generation cohort participants had sufficient serum 25(OH)D [25(OH)D > 20 ng/mL] and only 8.6 % and 1.2 % were considered to be at risk of inadequacy (<20 ng/mL) or deficiency (<12 ng/mL), respectively (thresholds defined by the IOM [3]). Given possible non-linear associations and/or threshold effects, our findings do not rule out a longitudinal vitamin D status—rate of change in pulmonary function association limited to persons at risk of inadequacy and/or deficiency.

Supporting this hypothesis, a recent study showed that individuals in the lowest quintile of serum 25(OH)D had a steeper rate of lung function decline compared with individuals in the highest quintile, where the mean in the highest quintile was 30 ng/mL [20]. In comparison, in our analysis of the Third Generation Framingham Heart Study cohort, the majority of the participants (>90 %) had serum 25(OH)D > 20 ng/mL. The recent finding of steeper decline limited to participants with the lowest serum 25(OH)D [20] and the lack of association in our analysis highlight the importance of considering non-linear associations and thresholds of effect defined by baseline nutritional status in future studies of 25(OH)D status and lung function.

A SNP in *CYP2R1* (rs11819875) was associated with rate of change in FEV_1_ in FHS, but did not show statistically significant evidence for replication of the association in a meta-analysis of four independent cohort studies (*P* = 0.09). However, the β-coefficient for this SNP was in the same direction in 3 of the 4 cohorts, and rs11819875 was associated with lower 25(OH)D in the SUNLIGHT consortium. The *P*-value for replication is near the threshold, and sample size, heterogeneity in environmental factors affecting usual vitamin D status (for example, sun exposure), and/or heterogeneity in the outcome measure (rate of change in FEV_1_) may contribute to the lack of replication. *CYP2R1* is a key hepatic 25-hydroxylase enzyme [38], and variants in this gene are consistently associated with 25(OH)D status in GWAS [5, 6]; this gene is a promising candidate for further studies investigating the role of vitamin D status in lung function phenotypes.

Our study has strengths and limitations. A limitation of the serum 25(OH)D—FEV_1_ analysis is that serum 25(OH)D was measured only once and a single measurement of vitamin D may not adequately estimate the long-term usual vitamin D status. We assumed that 25(OH)D measured at one point in time provided a reasonable approximation of 25(OH)D status throughout follow-up given that 25(OH)D measurements 12 months apart are highly correlated (r = 0.8 [39]) and given the relatively short follow-up period of 6–7 years. A related limitation is that supplement use data were not available, thus we could not investigate whether low serum 25(OH)D measures were associated with subsequent supplement use. While spirometry measurements in the Third Generation cohort were separated by an average of 6.1 years, a longer follow-up and more outcome measurements may be needed to identify associations between vitamin D and rate of change in FEV_1_. The Offspring participants included in the genetic analysis had an average spirometry follow-up of 14.7 years, leading to increased precision in the rate of decline estimates for the SNP—rate of change in FEV_1_ analysis. While the genetic analyses described in this study borrow concepts from the Mendelian Randomization (MR) literature [40], the limitations of the available data, including limited sample size, precluded a formal MR analysis. Overall, a major strength is the use of data from the Framingham Heart Study cohort, which provided a large, healthy, population-based sample including both men and women.

## Conclusions

The findings from this study suggest that serum 25(OH)D—FEV_1_ associations may be limited to individuals with inadequate 25(OH)D. Further, these findings suggest important study design considerations regarding potential to benefit from nutritional advice and/or intervention in future studies of 25(OH)D and lung function. Results of the SNP—FEV_1_ analysis demonstrate that genetic variants in a vitamin D metabolic gene are suggestively associated with rate of change in FEV_1_, and our results provide evidence to guide future studies.

## Additional file

Additional file 1:
**Table S1.** Imputed SNPs in Vitamin D Metabolic Genes in FHS. **Table S2.** Replication cohort associations of the most significant SNPs per gene with the rate of change in FEV_1_.
