# Supplementary material for: Association of 25-Hydroxyvitamin D status and genetic variation in the vitamin D metabolic pathway with FEV1 in the Framingham Heart Study
Source: Respir Res. 2015 Jul 1;16(1):81. doi: 10.1186/s12931-015-0238-y (PMC4491260; doi:10.1186/s12931-015-0238-y)
Supplement: Additional file 1: — Table S1. Imputed SNPs in Vitamin D Metabolic Genes in FHS. Table S2. Replication cohort associations of the most significant SNPs per gene with the rate of change in FEV1. [file 12931_2015_238_MOESM1_ESM.docx]

**Association of 25-Hydroxyvitamin D status and genetic variation in the vitamin D metabolic pathway with FEV_1_ in the Framingham Heart Study**

JG Hansen^1^ (jg553@cornell.edu), W Gao^2^ (gaowei@bu.edu), J Dupuis^2,3^ (dupuis@bu.edu), GT O’Connor^3,4^ (goconnor@bu.edu), W Tang^1^ (wt227@cornell.edu), M Kowgier^5,6^ (mkowgier@uhnresearch.ca), A Sood^7^ (ASood@salud.unm.edu), SA Gharib^8^ (sagharib@u.washington.edu), LJ Palmer^9^ (lyle.palmer@adelaide.edu.au) M Fornage^10,11^ (Myriam.Fornage@uth.tmc.edu), SR Heckbert^12,13,14^ (heckbert@u.washington.edu), BM Psaty^12,3,14,15^ (psaty@u.washington.edu), S Booth^16^ (sarah.booth@tufts.edu), SUNLIGHT^17^ and PA Cassano ^1,18^ (pac6@cornell.edu)

^1^ Division of Nutritional Sciences, Cornell University, 209 Savage Hall, Ithaca, NY 14853, USA

^2^ Department of Biostatistics, Boston University School of Public Health, Boston, MA, USA

^3^ The National Heart, Lung, and Blood Institute’s Framingham Heart Study, Framingham, MA, USA

^4^ Pulmonary Center, Department of Medicine, Boston University School of Medicine, Boston, MA, USA

^5^ Ontario Institute for Cancer Research, Toronto, ON, Canada

^6^ Dalla Lana School of Public Health, University of Toronto, Toronto, ON, Canada

^7^ University of New Mexico, Albuquerque, NM, USA

^8^ Computational Medicine Core, Center for Lung Biology, Division of Pulmonary & Critical Care Medicine, Department of Medicine, University of Washington, Seattle, WA, USA

^9^ School of Public Health, University of Adelaide, Adelaide, Australia

^10^ Institute of Molecular Medicine, University of Texas Health Science Center at Houston, Houston, TX, USA

^11^ Human Genetics Center, School of Public Health, University of Texas Health Science Center at Houston, Houston, TX, USA

^12^ Cardiovascular Health Research Unit, University of Washington, Seattle, WA, USA

^13^ Department of Epidemiology, University of Washington, Seattle, WA, USA

^14^ Group Health Research Institute, Group Health Cooperative, Seattle, WA, USA

^15^ Department of Medicine, University of Washington, Seattle, WA, USA

^16^ Jean Mayer USDA Human Nutrition Research Center on Aging, Tufts University, Boston, MA, USA

^17^ The SUNLIGHT Consortium (Study of Underlying Genetic Determinants of Vitamin D and Highly Related Traits)

^18^ Division of Biostatistics and Epidemiology, Department of Healthcare Policy and Research, Weill Cornell Medical College, New York, NY, USA

**ONLINE SUPPLEMENT**

**Supplemental Methods**

*Measures*

In the Offspring Exams 5, 6, and 7 spirometry was performed using a Collins Survey II spirometer (Collins Medical, Inc. Braintree, MA) calibrated daily and connected to a computer running software developed by S&M Instruments, Doylestown, PA [[1](#_ENREF_1), [2](#_ENREF_2)]. For Offspring Exam 8 and Third Generation Exams 1 and 2, spirometry was performed using a Collins CPL system (nSpire Health Inc., Longmont, CO) calibrated daily [[2](#_ENREF_2)]. Spirometry testing was performed in accordance with contemporaneous American Thoracic Society/European Respiratory Society standards [[3](#_ENREF_3)]. In both Offspring and 3rd Generation cohorts, a minimum of three spirometry tests were performed. Pulmonary function testing continued until three acceptable spirometry maneuvers meeting reproducibility criteria were obtained, up to a maximum of 8 maneuvers.

Serum 25(OH)D was assayed separately in the Offspring and Third Generation cohort serum samples using radioimmunoassay (RIA) (DiaSorin Inc, Stillwater, MN, USA) [[4](#_ENREF_4), [5](#_ENREF_5)], and log-transformed values were used in all analyses. In the Offspring cohort, the coefficient of variation (CV) for the serum 25(OH)D control values of 36 and 137 nmol/L were 8.5 and 13.2%, respectively [[5](#_ENREF_5)]. In the Third Generation cohort, the CV was 12.5% [[6](#_ENREF_6)]. Offspring serum samples for 25(OH)D assays were collected between 1997-2001 [[5](#_ENREF_5)] and Third Generation serum samples between 2001-2005 [[4](#_ENREF_4)]. The DiaSorin RIA assay was reformulated in 1998; however, all FHS samples were analyzed after 1998, so assay drifts due to the reformulated RIA assay, described for the NHANES data [[7](#_ENREF_7)], do not affect 25(OH)D measurements in Framingham. In addition to the RIA assay reformulation, drifts in assay performance were noted in NHANES, affecting the comparability of NHANES 25(OH)D measurements between 2003-2006. While it is not known if similar assay variation affects comparability between FHS Offspring and Third Generation serum 25(OH)D measurements, the effect of the assay drift was relatively small (statistical adjustment of mean 25(OH)D for assay drift in NHANES 2003-2004 and 2005-2006 resulted in mean 25(OH)D differences of 1-2 ng/mL [[8](#_ENREF_8)]).

*Statistical Analysis*

In serum 25(OH)D—rate of change in FEV_1_ analyses, the coefficient of interest was the interaction of 25(OH)D x time (time=time elapsed between the initial FEV_1_ measurement and each subsequent measurement), which estimated the effect of serum 25(OH)D on rate of change in FEV_1_. The cross-sectional association of serum 25(OH)D with FEV_1_ was estimated by the coefficient for serum 25(OH)D from the mixed effect models. The cross-sectional time point refers to the time point when serum 25(OH)D data was collected and assayed, not the date when the cohort study began. Smoothing spline analyses evaluated log-transformed 25(OH)D by residual FEV_1_, to examine linearity of the association. In SNP—rate of change in FEV_1_ analyses, the coefficient for the interaction of SNP x time estimated the additive effect of the coded allele on rate of change in FEV_1_. Baseline measurements for the Offspring participants included in the 25(OH)D—FEV_1_ analyses refer to measurements from the exam closest in time to the vitamin D measurement (either Exam 6 or 7); baseline measurements for Offspring participants in SNP—FEV_1_ analysis refer to Exam 5. Baseline measurements for the Third Generation participants refer to Exam 1. Serum 25(OH)D—FEV_1_ models were further adjusted for month of 25(OH)D measurement, body mass index (BMI) [[9](#_ENREF_9)], and FHS cohort (for models including both Offspring and Third Generation participants). Genetic models were further adjusted for the first two ancestry principal components to account for population substructure [[10](#_ENREF_10)].

*Replication Cohorts*

Three cohorts from the CHARGE (Cohorts for Heart and Aging Research in Genomic Epidemiology) consortium and one cohort from the SpiroMeta consortium were used for replication of the SNP— rate of change in FEV_1_ findings in the Framingham Heart Study. Cohorts included for the replication had ≥ 3 FEV_1_ measurements per participant, namely the Busselton Health Study (BHS), the Coronary Artery Risk Development in Young Adults (CARDIA), the Cardiovascular Health Study (CHS), the Health, Aging, and Body Composition Study (HABC), and the Framingham Heart Study (FHS). Further details on each cohort provided elsewhere [[11](#_ENREF_11)]. The combined replication cohorts included a total of 10,476 participants and 32,054 spirometry observations. Serum 25(OH)D data were not available for all replication cohorts, thus replication analyses were limited to SNP—FEV_1_ analyses.

**Supplemental Results**

*Association of 25(OH)D with Rate of Change in FEV_1_ in the Offspring Cohort*

In longitudinal analyses of 25(OH)D with rate of change in FEV_1_ , we observed a statistically significant interaction of cohort by time such that the Offspring cohort had a significantly attenuated rate of FEV_1_ decline compared to the younger Third Generation cohort. We further explored this finding in two sensitivity analyses. First, we excluded Offspring participants with COPD, defined as GOLD stages 1-4 (222 participants excluded), but the cohort x time interaction remained highly statistically significant (*P=*1.74x10^-25^). Second, we excluded all Offspring Exam 8 spirometry measurements given there was a change in the type of spirometer used at Exam 8 (186 participants excluded); however, once again the cohort x time interaction remained highly statistically significant in this analysis as well (*P=*2.32x10^-30^).

We hypothesize that the attenuated rate of decline in the Offspring compared to the Third Generation participants reflects a “healthy survivor” bias in the 1,435 Offspring cohort participants with serum 25(OH)D data, leading to systematic differences in rate of decline between cohorts. A previously published study examining FEV_1_ change in the Framingham Offspring cohort from Exams 1-6 demonstrated that the oldest male participants had a slight increase in FEV_1_, which the authors attributed either to healthy survivor bias or measurement variability [[1](#_ENREF_1)]. Due to the strong evidence for survivor bias, the Offspring cohort members with serum vitamin D did not contribute to analyses estimating the association of baseline serum vitamin D with subsequent change in FEV_1_**.**

**Table S1** Imputed SNPs in Vitamin D Metabolic Genes in FHS

| Gene Symbol | Chr | Function | Imputed SNPs in FHS |
| --- | --- | --- | --- |
| *CYP24A1* | 20 | Degradation of 1,25(OH)_2_D | 42 |
| *CYP27A1* | 2 | Vitamin D 25-hydroxylase | 23 |
| *CYP27B1* | 12 | 25(OH)D 1-α-hyroxylase | 5 |
| *CYP2R1* | 11 | Vitamin D 25-hydroxylase | 15 |
| *DHCR7/NADSYN1* | 11 | *DHCR7* converts vitamin D_3_ substrate to cholesterol; *NADSYN1* is a flanking gene | 105 |
| *GC* | 4 | Vitamin D binding protein | 51 |

**Table S2** Replication cohort associations of the most significant SNPs per gene with the rate of change in FEV_1_

| SNP | Gene | Coded Allele | BHS (N=1,009) | | | CARDIA (N=1,492) | | | CHS (3,159) | | | Health ABC (N=1,586) | | |
| --- | --- | --- | --- | --- | --- | --- | --- | --- | --- | --- | --- | --- | --- | --- |
|  |  |  | β | SE | *P* | β | SE | *P* | β | SE | *P* | β | SE | *P* |
| rs10877013 | *CYP27B1* | T | -1.2 | 1.1 | 0.2574 | -0.2 | 0.6 | 0.6941 | 1.1 | 1.2 | 0.3751 | -1.5 | 1.2 | 0.2117 |
| rs11819875 | *CYP2R1* | G | -1.1 | 1.4 | 0.4279 | -0.2 | 0.7 | 0.7924 | 0.2 | 1.5 | 0.9188 | -5.0 | 1.5 | 0.0006 |

Abbreviations: BHS = Busselton Health Study; CARDIA = Coronary Artery Risk Development in Young Adults Study; CHS = Cardiovascular Health Study; Health ABC = Health, Aging, and Body Composition Study; SNP = single nucleotide polymorphism; β = beta coefficient for SNP x time effect; SE = standard error; *P* = P-value

Adjusted for: baseline age, gender, height, smoking pattern over follow-up and its interaction with time, baseline smoking pack-years, and the first two principal components for genetic ancestry

**References**

1. Kohansal R, Martinez-Camblor P, Agusti A, Buist AS, Mannino DM, Soriano JB: **The natural history of chronic airflow obstruction revisited: an analysis of the Framingham offspring cohort.** *Am J Respir Crit Care Med* 2009, **180:**3-10.

2. Hancock DB, Eijgelsheim M, Wilk JB, Gharib SA, Loehr LR, Marciante KD, Franceschini N, van Durme YM, Chen TH, Barr RG, et al: **Meta-analyses of genome-wide association studies identify multiple loci associated with pulmonary function.** *Nat Genet* 2010, **42:**45-52.

3. Miller MR, Hankinson J, Brusasco V, Burgos F, Casaburi R, Coates A, Crapo R, Enright P, van der Grinten CP, Gustafsson P, et al: **Standardisation of spirometry.** *Eur Respir J* 2005, **26:**319-338.

4. Wang TJ, Zhang F, Richards JB, Kestenbaum B, van Meurs JB, Berry D, Kiel DP, Streeten EA, Ohlsson C, Koller DL, et al: **Common genetic determinants of vitamin D insufficiency: a genome-wide association study.** *Lancet* 2010, **376:**180-188.

5. Shea MK, Benjamin EJ, Dupuis J, Massaro JM, Jacques PF, D'Agostino RB, Sr., Ordovas JM, O'Donnell CJ, Dawson-Hughes B, Vasan RS, Booth SL: **Genetic and non-genetic correlates of vitamins K and D.** *Eur J Clin Nutr* 2009, **63:**458-464.

6. Cheng S, Massaro JM, Fox CS, Larson MG, Keyes MJ, McCabe EL, Robins SJ, O'Donnell CJ, Hoffmann U, Jacques PF, et al: **Adiposity, cardiometabolic risk, and vitamin D status: the Framingham Heart Study.** *Diabetes* 2010, **59:**242-248.

7. Yetley EA, Pfeiffer CM, Schleicher RL, Phinney KW, Lacher DA, Christakos S, Eckfeldt JH, Fleet JC, Howard G, Hoofnagle AN, et al: **NHANES monitoring of serum 25-hydroxyvitamin D: a roundtable summary.** *J Nutr* 2010, **140:**2030S-2045S.

8. CDC: **Revised Analytical Note for NHANES 2000-2006 and NHANES III (1998-1994) 25-Hydroxyvitamin D Analysis.** 2010.

9. Looker AC, Pfeiffer CM, Lacher DA, Schleicher RL, Picciano MF, Yetley EA: **Serum 25-hydroxyvitamin D status of the US population: 1988-1994 compared with 2000-2004.** *Am J Clin Nutr* 2008, **88:**1519-1527.

10. Price AL, Patterson NJ, Plenge RM, Weinblatt ME, Shadick NA, Reich D: **Principal components analysis corrects for stratification in genome-wide association studies.** *Nat Genet* 2006, **38:**904-909.

11. Tang W, Kowgier M, Loth DW, Soler Artigas M, Joubert BR, Hodge E, Gharib SA, Smith AV, Ruczinski I, Gudnason V, et al: **Large-scale genome-wide association studies and meta-analyses of longitudinal change in adult lung function.** *PLoS One* 2014, **9:**e100776.
